# Laparoscopic Surgery for Pediatric Malignant Ovarian Germ Cell Tumors After Neoadjuvant Chemotherapy: A Single-Center Retrospective Study

**DOI:** 10.3390/jcm15145366

**Published:** 2026-07-09

**Authors:** Jiabin Cai, Jieni Xiong, Xuan Wu, Min He, Lifeng Zhang, Dongfang Lu, Junqing Mao, Linjie Li, Yinbing Tang, Zheyu Ding, Ming Liu, Li Hou, Yuwei Wang, Qi Wang, Jinwen Meng, Ming Chen, Jinhu Wang

**Affiliations:** 1 Department of Oncology Surgery, Children’s Hospital, Zhejiang University School of Medicine, Hangzhou 310000, China; jiabincai@zju.edu.cn (J.C.);; 2 Department of Oncology Surgery, Children’s Hospital, National Clinical Research Center for Children and Adolescents’ Health and Diseases, Zhejiang University School of Medicine, Hangzhou 310052, China

**Keywords:** malignant ovarian germ cell tumors, neoadjuvant chemotherapy, laparoscopic surgery, pediatric oncology, minimally invasive surgery (MIS)

## Abstract

**Purpose**: The purpose of this study was to evaluate the clinical effects and safety for cancer treatment of neoadjuvant chemotherapy (NACT) when combined with laparoscopic fertility-sparing (anatomical preservation) surgery in pediatric patients who have large malignant ovarian germ cell tumors (MOGCTs). **Methods**: We performed a retrospective analysis on seven young female patients with a median age of 8.8 years (range: 10 months to 13.4 years). The patients were treated between 2015 and 2023, and all of them received two to three cycles of JEB (carboplatin, etoposide, and bleomycin) regimen as NACT. After the NACT, these patients had laparoscopic (n = 7) unilateral salpingo-oophorectomy. **Results**: Neoadjuvant chemotherapy (NACT) achieved a median reduction in tumor volume of 91% (range: 85% to 97%). All the procedures were completed successfully and there was no need to convert to open surgery. The median operative time was 85 min (range: 70 to 120 min), and the blood loss was minimal. The median blood loss was 10 mL, with a range of 5 to 50 mL. In patients with yolk sac tumors, alpha-fetoprotein (AFP) levels returned to normal within a median of 20 days after the operation. With a median follow-up of 54 months (range: 18 to 118 months), all the patients had 100% disease-free survival. **Conclusions**: This study suggests that NACT combined with laparoscopic fertility-sparing (anatomical preservation) surgery may be effective and appears promising for selected pediatric patients with MOGCTs.

## 1. Introduction

Malignant ovarian germ cell tumors (MOGCTs) are a rare type of ovarian cancer that mainly affects young girls and teenagers. Even though these tumors do not happen very often, they can cause significant clinical problems. They are very aggressive and can have a long-term impact on a girl’s physical and mental health, especially regarding their ability to have children. The problems associated with the tumor do not solely affect the patient themselves; they also cause a significant amount of emotional pain for their family. MOGCTs also have a serious impact on the economies of healthcare systems around the world. According to the data on how often these diseases occur, most ovarian cancers affect older women, but MOGCTs make up a substantial part of ovarian cancers in younger girls [1]. This shows that we need different treatment methods and support systems for different age groups.

Currently, the common ways to manage MOGCTs include a mixture of surgical removal and systemic chemotherapy. Platinum-based chemotherapy regimens are highly effective due to the sensitivity of these tumors [2]. With long-term survival rates for pediatric MOGCTs now exceeding 90% due to platinum-based regimens, the clinical focus has increasingly shifted from merely an oncologic cure to minimizing treatment-related morbidities and preserving long-term quality of life. Among these, ovarian follicle depletion and subsequent premature ovarian insufficiency (POI) induced by gonadotoxic chemotherapy represent profound challenges for young survivors. In the emerging field of pediatric oncofertility, achieving anatomical preservation of the contralateral ovary and uterus via fertility-sparing surgery is no longer the sole objective; rather, it serves as a critical structural baseline. For prepubertal and adolescent girls, safeguarding the remaining primordial follicle pool and exploring proactive fertility preservation pathways—such as ovarian tissue cryopreservation (OTC)—have become integral components of comprehensive, multidisciplinary pediatric cancer care [1,3,4]. Therefore, there is an important need in clinical practice to improve treatment methods. These improved methods should still work well against cancer, while also causing less physical harm and protecting the gonads [5,6].

To address these challenges, the combination of neoadjuvant chemotherapy (NACT) and minimally invasive surgery has gained traction. NACT offers the dual benefit of significant tumor downsizing and facilitating subsequent laparoscopic resection, thereby minimizing mechanical tissue trauma and maximizing the protection of the delicate adnexal vasculature. However, the literature documenting the intersection of NACT, laparoscopy, and modern pediatric fertility preservation strategies remains scarce. Therefore, this study aims to evaluate the oncologic and perioperative outcomes of a highly selected cohort of seven pediatric MOGCT patients managed with NACT followed by laparoscopic fertility-sparing (anatomical preservation) surgery. Through this, we seek to investigate the clinical feasibility of this approach and frame it within the evolving paradigm of contemporary pediatric gonadal and reproductive preservation. Previous studies have shown that NACT can effectively make the tumor smaller, improves the possibility of fertility-sparing (anatomical preservation) procedures, and may improve survival results [7,8]. At the same time, the development and improvement of minimally invasive surgical methods, especially laparoscopic surgery, have obvious advantages. These include less surgical injury, less blood loss, shorter hospital stays, and faster recovery. These benefits are especially important in pediatric oncology. In this field, surgical risks must be carefully considered with regard to long-term quality of life [9,10]. However, the combination of NACT and laparoscopic fertility-preserving surgery in treating pediatric MOGCTs has not been sufficiently studied. There is limited data on safety and efficacy, and we have noticed that there is a lack of knowledge in this area. Therefore, the current study focuses on using neoadjuvant chemotherapy (NACT) and laparoscopic surgery together. This treatment is designed for young female patients diagnosed with malignant ovarian germ cell tumors to retain their ability to have children. We looked back at the clinical results of seven young patients who were treated between 2015 and 2023. Our goal was to figure out how well this combined treatment works, how safe it is, and how it affects patients’ recovery after the operation. By looking at these real-world cases, we can obtain useful information about whether this treatment is practical. This can also provide a foundation for future studies.

The main goal of this study is to see how well NACT, followed by laparoscopic fertility-sparing (anatomical preservation) surgery, works to control tumors. At the same time, it aims to reduce surgical problems in pediatric patients with MOGCTs. The secondary goals are to look at how patients recover after the operation and what complications they might have. This helps in coming up with better treatment plans that focus on both treating cancer and retaining fertility. In this study we will analyze a significant amount of clinical data, aiming to provide evidence-based suggestions and improve the way we treat this group of patients.

## 2. Materials and Methods

### 2.1. Patient Population

This was a retrospective, single-center cohort study. It included patients who were diagnosed with MOGCTs at the Department of Oncology Surgery, Children’s Hospital, Zhejiang University School of Medicine, from 2015 to 2023. The patients were consecutive cases. During this period, there were 34 patients diagnosed as MOGCTs in total, and 27 patients underwent a radical surgery after diagnosed. NACT was indicated for patients with massive tumors (defined as those with a diameter > 10 cm or the upper border of the tumor extends beyond the umbilical level.) and those with high risk of surgical rupture spillage, and 7 patients were selected for NACT based on these criteria.

The patients first received two to three cycles of NACT before surgery. After neoadjuvant chemotherapy, the patients underwent laparoscopic unilateral salpingo-oophorectomy. The final pathological result showed that they had malignant ovarian germ cell tumors (MOGCTs). The final cohort comprised seven patients.

### 2.2. Staging

The staging followed the Children’s Oncology Group (COG) criteria. Six patients were classified as Stage I. One patient had preoperative imaging evidence of localized rupture, and this patient was classified as Stage II. The patients’ information is summarized in Table 1. The tumor volume was calculated using the ellipsoid formula (0.5 × length × width × height) based on CT or MRI measurements.

### 2.3. Protocol

All patients were suspected of having malignant ovarian germ cell tumors after admission. Apart from one patient who showed signs of tumor rupture, the remaining six patients underwent ultrasound-guided core-needle tumor biopsy. For the patient with tumor rupture at presentation, the diagnosis was established based on characteristic imaging findings (CT/MRI) and elevated serum tumor markers (AFP: 138,865 ng/mL), obviating the need for biopsy to avoid further tumor spillage. Postoperative pathology confirmed malignant ovarian germ cell tumors in all cases. All patients received two to three cycles of a JEB (carboplatin, etoposide, and bleomycin) chemotherapy regimen [11,12]. The neoadjuvant JEB regimen was administered intravenously in 21-day cycles. The standardized dosages were as follows: carboplatin 600 mg/m^2^ (day 1), etoposide 120 mg/m^2^/day (days 1–3), and bleomycin 15 units/m^2^ (day 1). For infants under 12 months of age, dosages were adjusted based on body weight (mg/kg): Body weight dose = Surface area dose/30 × Body weight (kg). Patients received two to three cycles of NACT before surgical reassessment.

### 2.4. Surgical Procedure and Laparoscopic Approach

All of the procedures were performed by the same group of experienced pediatric surgeons. They used a three-port laparoscopic approach. After neoadjuvant chemotherapy (NACT), the tumors were still too large. To stop the tumors from rupturing, the surgeons strictly followed a “no-touch” technique when moving the tumors. The tumors had shrunk significantly (the median reduction was 91%) and had more fibrotic capsules after chemotherapy. The surgeons carefully cut the tumors away from the surrounding tissues. They removed the tumor, along with the ovary and the fallopian tube on the same side all together. To prevent the tumor from spilling during the operation and contaminating the peritoneum, they put the removed specimen into an endoscopic retrieval bag right away in the pelvic cavity. For large specimens, they protected them in the bag and took them out through an extended umbilical incision. No intraoperative tumor spillage occurred in any of the seven cases. The ‘no-touch’ technique and use of endoscopic retrieval bags ensured intact specimen removal in all cases.

### 2.5. Statistical Analysis

Descriptive statistics were used to summarize the clinical data. Continuous variables were expressed as median (range), depending on the distribution. Categorical variables were presented as frequencies and percentages. Due to the small sample size (n = 7), no comparative hypothesis testing was performed. All statistical analyses were conducted using SPSS version 26.0.

## 3. Results

### 3.1. General Data and Pathological Characteristics

A total of seven pediatric patients were enrolled in this study, with ages ranging from 10 months to 13 years and 5 months (median age: 8.8 years). Among them, one case was under 1 year old, three cases were aged 1–10 years, and three cases were over 10 years old. The pathological types included three cases of endodermal sinus tumor (yolk sac tumor), two cases of dysgerminoma, and two cases of malignant germ cell tumor (mixed type).

### 3.2. Response to NACT

**Figure 1:**
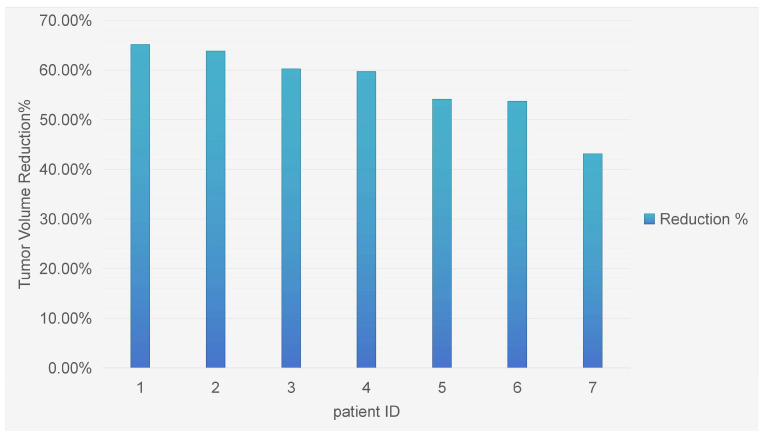
Waterfall plot of maximal tumor volume changes after neoadjuvant chemotherapy (NACT).

The median maximum tumor diameter before chemotherapy was 13.0 cm (range 8.0–21.0 cm (Table 1). After NACT, the maximum tumor diameter before surgery decreased to 3.5 cm to 7.6 cm, with an average maximum diameter of (5.4 ± 1.4) cm (Table 2, Figure 1).

**Figure 2:**
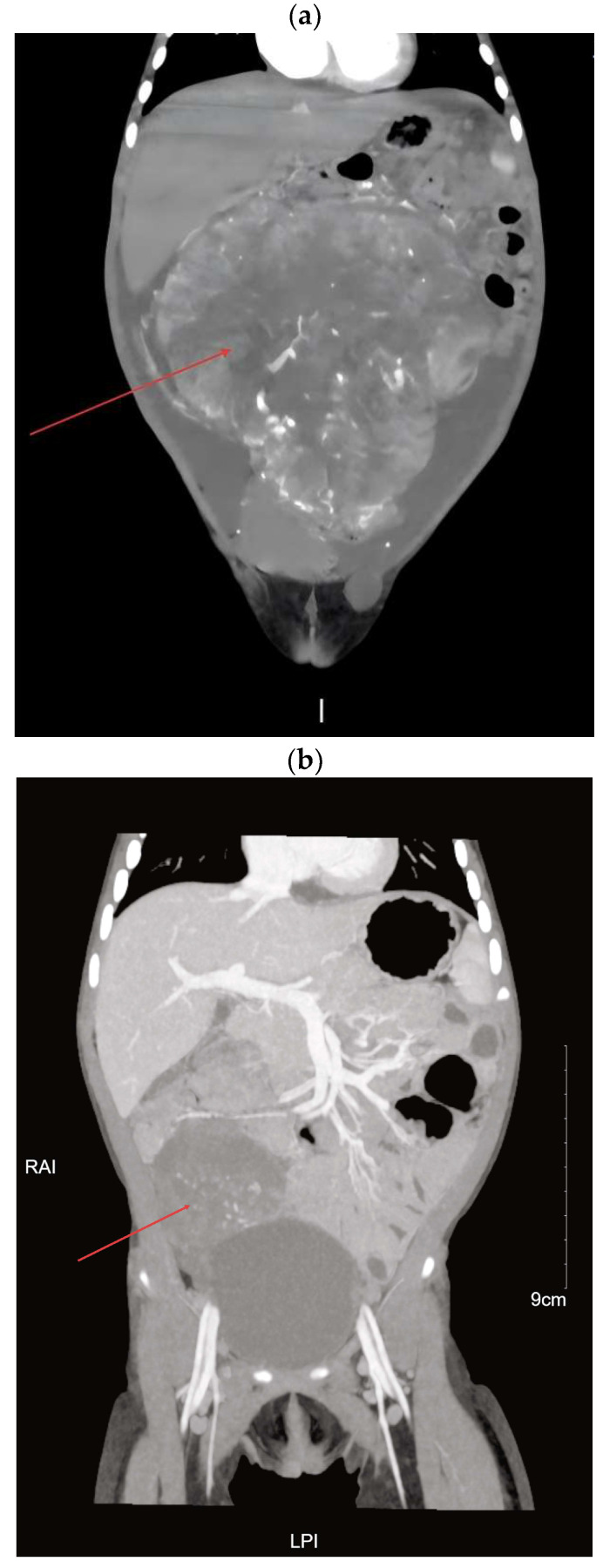
Enhanced abdomen CT demonstrates changes in tumor size before and after chemotherapy, and the arrow indicates the tumor before and after chemotherapy: (**a**) tumor before chemotherapy; (**b**) tumor after two cycles of chemotherapy.

The median tumor volume decreased significantly from 1609 cm^3^ to 144 cm^3^, representing a 91% reduction. A 21 cm tumor was successfully downsized to 7.6 cm after three cycles. Abdominal contrast-enhanced CT images showing tumor changes in a patient before and after two cycles of chemotherapy (Figure 2). The median plasma level of the tumor marker (AFP) in patients with yolk sac tumors decreased from 137,403 ng/mL to 534 ng/mL, representing a 99.9% reduction.

Each bar represents an individual patient (n = 7). The vertical axis shows the percentage change in the maximal tumor diameter from baseline to the preoperative assessment following two to three cycles of JEB chemotherapy. All patients achieved a significant reduction in tumor burden. The median reduction rate was 59.7%. This made it easier to perform the subsequent minimally invasive surgical resection.

### 3.3. Surgical and Perioperative Outcomes

All seven cases in the group had successful minimally invasive surgery. There were no complications during the operation, and the surgeons did not need to switch to laparotomy. The clinical data and perioperative outcomes are summarized in Table 3.

### 3.4. Recovery and Follow-Up

Postoperative chemotherapy: The pediatric patients recovered well after the surgery. No delays in starting postoperative chemotherapy were attributable to surgical complications. The median time to start postoperative chemotherapy was 7.1 days (range 6–11 days), with all patients recovering uneventfully from surgery. The serum AFP levels returned to normal within a median of 20 days before the next chemotherapy cycle. All patients finished the whole JEB-regimen chemotherapy. The treatment period was between 2015 and 2023.

Prognostic follow-up: No patients were lost to follow-up. All seven patients completed the follow-up period, with a median duration of 54 months (range 18–118 months). The follow-up results were good, and there were no signs of tumor recurrence or metastasis.

During the preparation of this manuscript, the authors used Gemini 3.0 for the purpose of language translation.

## 4. Discussion

Malignant ovarian germ cell tumors (MOGCTs) are a rare but important type of pediatric ovarian tumors. They are known for being aggressive and can have a long-term impact on patients’ reproductive health. These tumors make up a small part of ovarian cancers in children and adolescents. When diagnosing them, we need to think carefully, because we are faced with two challenges: controlling the cancer effectively and preserving the patients’ future fertility.

Since MOGCTs are rare, it is challenging to set up standard treatment plans. Patients are forced to deal with the physical problems of the disease, but they also face psychosocial stress, and their families and the healthcare system have to deal with economic issues. These tumors are very sensitive to chemotherapy. In pediatric oncology, it is important to balance the strength of the treatment and reduce the side-effects related to the treatment.

In this study, we try to solve these challenges. We do this by looking back at seven young female patients who were diagnosed with MOGCTs. The study focuses on using neoadjuvant chemotherapy (NACT) first and then performing minimally invasive laparoscopic surgery to retain the patients’ fertility. In this study, we use real-world clinical data to give us an idea of how well and how safely NACT can be combined with surgery that retains fertility in these young patients. The study points out some important findings. After NACT, the tumors were significantly reduced. The doctors were able to perform the laparoscopic surgery successfully many times without any problems during the operations. In addition, the patients had good long-term results in terms of disease-free survival.

In our group of patients, we saw a sizeable reduction in the volume of solid tumors after neoadjuvant chemotherapy (NACT). The median decrease was 91%. This fits with what we know about how sensitive malignant ovarian germ cell tumors (MOGCTs) are to platinum-based treatment plans, especially the JEB protocol. This large decrease in volume most likely shows the toxic effects of carboplatin, etoposide, and bleomycin on fast-growing germ cell groups. These drugs can cause the cells to die and stop them from copying their DNA [13]. Platinum drugs make the DNA strands stick together, which stops the cell cycle and causes the cells to die. Etoposide stops an enzyme called topoisomerase II, which breaks the DNA strands. Bleomycin can generate free radicals, and these free radicals cause DNA damage. This kind of multiple-pathway cell-killing effect works to enhance the killing of tumor cells. Previous studies have mainly focused on the role of NACT in removing epithelial ovarian cancer, while our findings show that similar ideas can also be used in pediatric MOGCTs [14]. The main difference is that we focus on protecting fertility. The tumor volume is significantly reduced using this treatment, and this reduction provides enough space for laparoscopic surgery. As a result, there are more opportunities for minimally invasive operations. It is worth noting that when the tumor shrinks significantly, this can reduce the tumor burden. This makes minimally invasive surgical methods easier to use, and also reduces the risk during the operation. The change in tumor volume in our study is different from what was reported in mature cystic teratoma malignancies [15]. In those cases, the effect of chemotherapy is not always the same. Our data prove that the JEB treatment plan is useful for pediatric MOGCTs. It provides new evidence that this treatment can help patients be better candidates for surgery by effectively reducing the tumor.

One of the main concerns about laparoscopic treatment of MOGCTs is the risk of iatrogenic rupture and then peritoneal seeding. The tumors shrank significantly (the median reduction was 91%) and developed more fibrotic capsules after chemotherapy. For Case 6 (ruptured tumor at presentation), NACT resulted in a 93% tumor volume reduction and complete encapsulation of the tumor, with no evidence of residual rupture or ascites on post-NACT imaging. Surgical exploration confirmed a well-encapsulated mass without peritoneal contamination. This makes it possible to safely remove the tumor through laparoscopic surgery.

We used a strict endobag extraction protocol to protect tumors from rupture and seeding. We used a retrieval bag and took out the specimen through an umbilical extension. This made sure that the tumor did not touch the abdominal wall at all. Also, the three-port technique caused less peritoneal trauma than open laparotomy. This enables patients to receive the next chemotherapy session earlier after surgery. (the median was POD 7.1).

Although a direct comparison with open surgery was not performed, the observed outcomes suggest that laparoscopic surgery after NACT may offer advantages in terms of reduced blood loss and shorter hospital stay, which warrants further investigation in controlled studies. This is because the tumor size is reduced and the surgical field can be seen more clearly. The minimally invasive method takes advantage of the tumor shrinkage caused by chemotherapy. It allows for precise tumor removal with little blood loss and less tissue damage. This is shown by the small amount of bleeding during the operation and the fact that there was no need to switch to an open laparotomy. Similar results have been found in advanced gastric and pancreatic cancers. When laparoscopic resection is performed after NACT, the cancer treatment results are similar, but patients recover more quickly [14,16]. Biologically, after chemotherapy, the tumor has fewer blood vessels and less scar-like tissue. This may lead to less bleeding during the operation and make it easier to remove the tumor all at once. In addition, laparoscopy lets doctors carefully check the peritoneal cavity, which helps in accurately staging the cancer and planning the resection [17].

Margin assessment is very important. This is because the status of residual disease has a significant influence on prognosis. Our study applies these ideas to pediatric MOGCTs. It shows that when we use neoadjuvant chemotherapy (NACT) and minimally invasive surgery together, it not only keeps patients safe from cancer but also helps keep their reproductive structures. This is better than the traditional laparotomy, which usually requires the removal of more tissue.

The safety profile we observed was very positive. There were very few perioperative complications, and the patients could follow the chemotherapy protocols without having to reduce the dose. This shows that when we combine NACT with laparoscopic surgery, it can reduce the treatment-related toxicity in pediatric MOGCTs. The reason behind this may be that the tumor burden is reduced and the operative stress is also lower. This can decrease the systemic inflammatory responses and postoperative immunosuppression, which are important for chemotherapy tolerance [18].

Moreover, the JEB chemotherapy regimen is quite powerful. It is also known that in germ cell tumors, it has a relatively manageable range of side-effects. The most common side-effects are hematological adverse events, but these can be controlled [13].

Minimally invasive surgery can preserve the anatomy related to fertility. This might also lower the chance of long-term endocrine dysfunction, which is a significant concern for pediatric patients [19]. This contrasts with data from high-grade serous ovarian carcinoma, where extensive, cytoreductive surgery may make the patient’s condition worse [20]. Our findings show that the combined treatment method has a good balance. It can make the treatment more effective and reduce harm at the same time. This is a good way to treat pediatric ovarian tumors.

The treatment strategy for pediatric MOGCTs is very different from that for adults. This difference is especially clear in terms of when to perform surgery and how important it is to preserve fertility. In adult patients, the usual method is to carry out primary cytoreductive surgery first. Neoadjuvant chemotherapy (NACT) is usually only for patients who cannot have surgery because their disease is at an advanced stage or they are not in good physical condition [21]. According to the COG guidelines (Billmire & Frazier), the optimal timing for surgery in pediatric MOGCT is to perform fertility-sparing radical surgery as soon as the diagnosis is confirmed. The paramount importance and feasibility of fertility-sparing are underpinned by an exceptionally high overall survival rate of 96%; this remarkable survival advantage empowers us to strive for a therapeutic goal where patients not only survive but also thrive with a high quality of life, including the ability to bear children [22,23]. In our study, we saw that the tumor volume decreased by a median of 91%. Due to the high chemosensitivity of the tumors, this means that NACT can be used as a way to make minimally invasive surgery easier, not just as a final option.

Adult treatment protocols for MOGCTs increasingly include fertility-sparing surgery (FSS). But in children, large tumors have anatomical constraints. In traditional situations, they often require extensive laparotomy. Our findings show that NACT-induced downsizing can solve this problem in pediatric cases. It can turn huge abdominal masses into smaller, more manageable lesions. These lesions are suitable for a three-port laparoscopic approach. In adult cases, high-grade serous carcinomas may respond differently to NACT [24]. However, pediatric yolk sac tumors and dysgerminomas shrink in volume more uniformly and significantly. This reduces the risks of intraoperative tumor spillage and the need to switch to laparotomy.

Furthermore, in pediatric oncology, the focus on long-term quality of life is more obvious. Adult survivors of MOGCTs also value fertility, but pediatric patients have to deal with decades of potential reproductive and endocrine health issues. In these cases, we implemented the JEB regimen and then performed laparoscopy. This approach not only ensures excellent 54-month disease-free survival, which is as good as the best adult outcomes, but it also reduces the physical and psychological problems related to large surgical scars and extensive tissue damage. This combined approach fits the changing trend in pediatric surgical oncology. In this trend, we try to reduce the intensity of surgical trauma without sacrificing oncological safety.

We observed sustained disease-free survival (DFS) over a median follow-up of 54 months. All patients remained recurrence-free. This shows the prognostic impact of achieving complete cytoreduction after neoadjuvant chemotherapy (NACT) and laparoscopic surgery. Mechanistically, this might be because the multimodal approach can effectively treat both macroscopic and microscopic diseases. Residual tumor burden is a well-known predictor of relapse in ovarian malignancies [25].

The AFP levels returned to normal within a median of 20 days after the operation. This shows strong evidence that our minimally invasive approach is effective in surgery. According to COG guidelines, the quick clearance of serum markers means there is no macroscopic or significant microscopic residual disease [26]. This fast serological remission, along with a 100% complete remission rate at the final follow-up in December 2025, suggests that the oncological outcomes of laparoscopic surgery after NACT are good.

Preserving reproductive function without sacrificing oncological outcomes shows the benefits of customized surgical strategies. These strategies are based on tumor biology and how the tumor responds to chemotherapy. Studies on adult patients have shown that better cytoreduction, especially with neoadjuvant chemotherapy (NACT), is linked to longer progression-free and overall survival [27]. In our cohort, all seven patients successfully underwent laparoscopic unilateral salpingo-oophorectomy without any damage to the contralateral adnexa. This demonstrates that laparoscopic surgery following NACT is highly feasible for achieving structural gonadal preservation. Crucially, in contemporary pediatric oncology, a conceptual distinction must be maintained between ‘fertility-sparing surgery’ (i.e., surgical gonadal preservation) and comprehensive ‘fertility preservation’ pathways. While the structural preservation of the contralateral ovary and uterus was achieved in our cohort, modern pediatric oncofertility paradigms emphasize proactive interventions, particularly for prepubertal girls for whom mature oocyte cryopreservation is biologically impossible. In such young cohorts, including the infants and toddlers presented in our study, ovarian tissue cryopreservation (OTC) currently represents the sole realistic and established strategy for safeguarding future reproductive potential [28]. One study recently demonstrated the robust long-term feasibility and safety of OTC in a two-decade registry encompassing 451 children and adolescents, underscoring its role as a clinical standard rather than an experimental modality. Furthermore, for malignancies such as MOGCTs, where concerns regarding the inadvertent reintroduction of occult malignant cells during future autologous tissue retransplantation persist, evolving technologies like in vitro growth (IVG) and in vitro maturation (IVM) from isolated primordial follicles offer critical future theoretical alternatives to mitigate oncologic risks.

Implementing successful pediatric OTC requires meticulous optimization of cryopreservation methodologies and regional healthcare logistics. While traditional slow freezing has long served as the historical baseline, vitrification (ultra-rapid cooling) has emerged as a superior or at least highly promising alternative, offering excellent preservation of primordial follicle structures. This is supported by landmark clinical evidence in Europe reporting a successful live birth following the retransplantation of vitrified-warmed ovarian tissue [29]. Given the extreme rarity of pediatric MOGCTs, a single-center approach to cryopreservation is often restricted by technical and volume limitations. Establishing a centralized cryobanking network—replicating the validated Copenhagen or Bonn models—is vital to standardizing tissue processing and quality control. Recent multi-center data validate that overnight transportation of harvested ovarian tissue at 4 °C to a centralized processing facility is biologically safe, maintaining high follicular viability and not compromising long-term reproductive outcomes within clinically applicable transport windows [30].

Our findings suggest that NACT combined with laparoscopy provides a viable minimally invasive approach for selected patients. We must candidly acknowledge several significant limitations inherent to this study. First, the retrospective, single-center design and the highly selective nature of the patient cohort inherently introduce selection bias. Second, our sample size is extraordinarily small (n = 7) and lacks a matched primary surgery control group, precluding any definitive or generalized claims regarding statistical safety and therapeutic superiority. Consequently, our findings must be interpreted strictly as preliminary and hypothesis-generating. Third, a major shortcoming is the total absence of long-term objective reproductive and endocrine follow-up data. Endpoints such as serial anti-Müllerian hormone (AMH) monitoring, pubertal developmental velocity, and long-term menstrual or pregnancy outcomes were not uniformly tracked. Thus, while the immediate oncologic clearance and perioperative profiles are encouraging, the definitive functional status of the preserved gonadal apparatus remains to be verified through prospective, international registries.

This study suggests that NACT combined with laparoscopic fertility-sparing (anatomical preservation) surgery may be effective and appears promising for selected pediatric patients with MOGCTs. However, these findings are preliminary and require validation in larger, prospective, multi-center studies. Future research should also explore the long-term effects on fertility and quality of life and improve treatment methods based on different patient demographics and tumor characteristics.

## Figures and Tables

**Table 1:** Patients’ basic information before chemotherapy.

Case	Age	Primary Pathology	Pre-NACT Size	COG Stage
1	6 years 11 months	Dysgerminoma	8.8 cm	**Stage I**
2	13 years 5 months	Dysgerminoma	21.0 cm	**Stage I**
3	8 years 10 months	Mixed MGCT	13.3 cm	**Stage I**
4	13 years 2 months	Mixed MGCT	12.6 cm	**Stage I**
5	9 years 8 months	Yolk Sac Tumor	14.9 cm	**Stage I**
6	10 months	Yolk Sac Tumor	12.9 cm	**Stage II**
7	1 year 10 months	Yolk Sac Tumor	10.2 cm	**Stage I**

MGCT: malignant ovarian germ cell tumors.

**Table 2:** Changes in the maximal tumor diameter before and after neoadjuvant chemotherapy.

Case No.	Diameter Pre-Chemotherapy (cm)	Diameter Post-Chemotherapy (cm)	Reduction (%)
Case 1	8.8	3.5	−60.20%
Case 2	21	7.6	−63.80%
Case 3	13.3	6.1	−54.10%
Case 4	12.6	4.4	−65.10%
Case 5	14.9	6.9	−53.70%
Case 6	12.9	5.2	−59.70%
Case 7	10.2	5.8	−43.10%

**Table 3:** Clinical and perioperative data of the cohort (n = 7).

Parameters	Median (Range)
Age	8.8 years (10 months–13.4 years)
Initial Tumor Volume	1609 cm^3^ (409–3780 cm^3^)
Tumor Volume Reduction Rate	91% (85–97%)
Operative Time	85.0 min (70–120 min)
Estimated Blood Loss	10.0 mL (5–50 mL)
Time to AFP Normalization	20.0 days (7–20 days)
Interval to Adjuvant Chemo	7.1 days (6–11 days)
Follow-up Duration	54.0 months (18–118 months)
Disease-free Survival (DFS)	100%

## Data Availability

The data presented in this study are available on request from the corresponding author.
